# Editorial: International Day of Persons with Disabilities – children's disabilities

**DOI:** 10.3389/fpubh.2024.1483318

**Published:** 2024-09-20

**Authors:** Olaf Kraus de Camargo, Nihad A. Almasri, Thorsten Langer

**Affiliations:** ^1^Faculty of Health Sciences, CanChild, McMaster University, Hamilton, ON, Canada; ^2^Division of Developmental Pediatrics, Department of Pediatrics, Faculty of Health Sciences, McMaster University, Hamilton, ON, Canada; ^3^Department of Rehabilitation Science, College of Health Sciences, Qatar University, Doha, Qatar; ^4^Department of Physiotherapy, School of Rehabilitation Sciences, The University of Jordan, Amman, Jordan; ^5^Department of Pediatrics, University of Freiburg, Freiburg im Breisgau, Germany

**Keywords:** inclusion, policies, epistemic (in)justice, low- and middle-income countries (LMIC), accessibility

As we observe the International Day of Persons with Disabilities, it is essential to reflect on the progress and ongoing challenges in supporting children with disabilities. As editors we were excited with the opportunity to gather the experience from researchers around the world and to see the significant interest generated by this Research Topic with over 36.000 views as of the date of this writing (https://www.frontiersin.org/research-topics/52675/international-day-of-persons-with-disabilities—childrens-disabilities/magazine).

The Research Topic of 24 articles featured in this publication provides a comprehensive overview of various issues, from educational access (Odeh and Lach and Campbell et al.) and healthcare needs (Muehlan et al.) to the impact of socioeconomic factors (O'Donnell et al., and Janus et al.) and the COVID-19 pandemic on children with disabilities and their families (Katalifos et al. and Pozniak et al.) to name a few. These studies underscore the importance of inclusive policies, coordinated support systems, and community-based interventions to ensure that no child is left behind.

A common thread running through these articles is the critical need for inclusive, equitable, and comprehensive policies and practices to support children with disabilities and their families. Whether addressing socioeconomic disparities, leveraging technological innovations, ensuring effective healthcare transitions, or providing robust educational support, the central theme is the importance of tailored, responsive, and integrated approaches that recognize the unique challenges faced by children with disabilities.

As we reflect on these studies, it is evident that while significant progress has been made in supporting children with disabilities, much work remains. The article of Materula et al. analyzing data from the province on Alberta in Canada gives a detailed overview what outcomes to measure to assess the variety of interventions and their effects on children, families and the support system as a whole.

Most authors of this Research Topic are from high-income countries, mostly from North America and Europe. This is concerning as low-and-middle income countries are the home of the majority of children living with disabilities (approximately 80%) (1, 2). Research and data are disproportionately scarce in LMICs, which contributes to a limited understanding of healthcare and educational services for people with disabilities (3). This imbalance in knowledge has significant implications for the development of inclusive policies and practices that can address the needs of people with disabilities in these countries.

As editors we tried to address the biases that could result in such an epistemic injustice by disseminating the call for submissions broadly in the different networks and contexts in which we work and inviting colleagues from LMICs to submit and review (4, 5). Nonetheless, there were also barriers out of our control such as limited availability of financial waivers of article processing charges and potential language barriers that limited submissions in English (6, 7).

Considering this background, we want to highlight five publications out of the 24 accepted that describe issues relevant to children with disabilities in LMICs:

Kaur et al. explore the experiences of families with young children with autism in Delhi during the COVID-19 pandemic. This study identifies significant impacts on family life, financial stability, and the wellbeing of children with autism. It emphasizes the need for flexible service delivery, remote interventions, and comprehensive support systems for caregivers to support such families in future crises.

López et al. examine whether the educational rights of children under five with disabilities are acknowledged and supported in Chile. The article highlights significant challenges in data collection, inter-agency coordination, and the practical implementation of policies supporting young children with disabilities. It calls for improved data collection, targeted policy measures, and increased awareness among families to ensure the educational rights of these children are fully supported.

The study of Morrison et al. underscores the need for contextually specific research to understand the experiences of adolescents with disabilities during pandemics and other emergencies by engaging with adolescents with disabilities in Nepal. It advocates for inclusive policies and support systems that address the intersecting vulnerabilities of disability, socio-economic status, and rural isolation to improve outcomes for this marginalized group.

Dada et al. present an opinion article discussing the challenges faced by newcomer children with disabilities and their families in Canada. Many of those newcomers are refugees or immigrants from LMICs. The article highlights significant barriers such as language, cultural differences, and financial constraints, calling for inclusive practices and policies to ensure these vulnerable populations receive the care and services they need to thrive.

Another study from China (Jacobs et al.), a high-middle-income country, highlighted the effectiveness of a large-scale vision impairment screening program for children with complex disabilities. As a result of this project, over 1.32 million children were screened, and more than 1,363 children with both complex disabilities and visual impairment were identified. The collaboration between healthcare providers and educators in China has led to significant improvements in diagnosing and supporting children with disabilities, and efforts to sustain these advances are now being championed by key government officials. The success of this project demonstrates the impact of focused intervention programs in LMICs and the crucial role of multi-sectoral collaboration, providing a model that other LMICs could adopt to address complex disabilities more effectively.

By addressing the gaps identified in these articles and implementing the recommended policies and practices, we can move closer to a world where every child, regardless of their abilities, has the opportunity to thrive. To further improve the contribution of researchers from LMICs to disability research we suggest the following strategies. First, there is a need for international research collaborations that actively include and support LMIC researchers, providing them with access to funding, mentorship, and publication opportunities. Encouraging the establishment of research networks that prioritize inclusive practices, such as capacity building and knowledge exchange and providing open access to journals and research databases. Policymakers and funding agencies should also recognize the importance of supporting disability-related research in LMICs, as addressing the unique challenges faced by individuals in these regions will have global benefits in promoting inclusive development.

This International Day of Persons with Disabilities serves as a reminder of our collective responsibility to ensure that no child is left behind—in every country. This applies to the medical, educational and social care work for persons with disabilities as well as the research and its associated regulations and policies alike.
